# Supplementary figures and images for: Testing methods to mitigate Caribbean yellow-band disease on Orbicella faveolata
Source: PeerJ. 2018 May 11;6:e4800. doi: 10.7717/peerj.4800 (PMC5951125; doi:10.7717/peerj.4800)

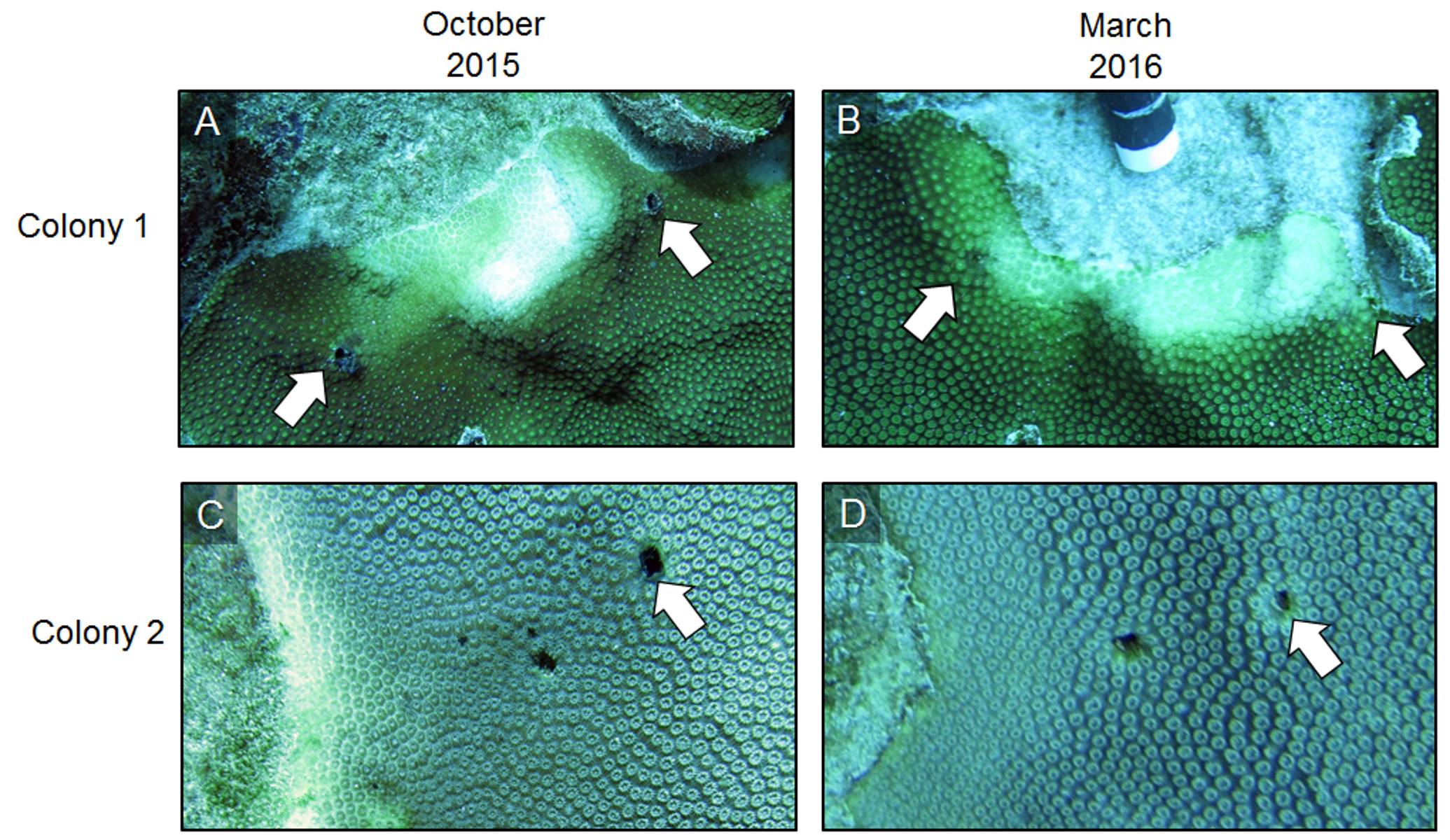

Supplement: Supplemental Information 1 — Time series images of two representative coral colonies immediately following the removal of masonry nails from shade cloth installation (October 2015) and five months post-removal (March 2016). Masonry nail holes are indicated by arrows. Photo credit: C. J. Randall. [file peerj-06-4800-s001.png]
